# ITS2 Secondary Structure Improves Discrimination between Medicinal “Mu Tong” Species when Using DNA Barcoding

**DOI:** 10.1371/journal.pone.0131185

**Published:** 2015-07-01

**Authors:** Wei Zhang, Yuan Yuan, Shuo Yang, Jianjun Huang, Luqi Huang

**Affiliations:** 1 Marine College, Shandong University at Weihai, Weihai, Shandong, China; 2 State Key Laboratory of Dao-di Herbs, National Resource Center for Chinese Materia Medica, China Academy of Chinese Medical Sciences, Beijing, China; Saint Mary's University, CANADA

## Abstract

DNA barcoding is a promising species identification method, but it has proved difficult to find a standardized DNA marker in plant. Although the ITS/ITS2 RNA transcript has been proposed as the core barcode for seed plants, it has been criticized for being too conserved in some species to provide enough information or too variable in some species to align it within the different taxa ranks. We selected 30 individuals, representing 16 species and four families, to explore whether ITS2 can successfully resolve species in terms of secondary structure. Secondary structure was predicted using Mfold software and sequence-structure was aligned by MARNA. RNAstat software transformed the secondary structures into 28 symbol code data for maximum parsimony (MP) analysis. The results showed that the ITS2 structures in our samples had a common four-helix folding type with some shared motifs. This conserved structure facilitated the alignment of ambiguous sequences from divergent families. The structure alignment yielded a MP tree, in which most topological relationships were congruent with the tree constructed using nucleotide sequence data. When the data was combined, we obtained a well-resolved and highly supported phylogeny, in which individuals of a same species were clustered together into a monophyletic group. As a result, the different species that are often referred to as the herb “Mu tong” were successfully identified using short fragments of 250 bp ITS2 sequences, together with their secondary structure. Thus our analysis strengthens the potential of ITS2 as a promising DNA barcode because it incorporates valuable secondary structure information that will help improve discrimination between species.

## Introduction

The internal transcribed spacer (ITS) region of rDNA is the most widely used phylogenetic marker and has contributed greatly to plant phylogeny and the whole life tree [1–3]. This region is composed of ITS1/ITS2 intergenic sequences with highly conserved 5.8 rRNA in between. ITS2, the principal source of ITS sequence variation, is shorter and easier to sequence than ITS, and therefore has been recognized as an excellent phylogenetic marker and a promising standardized region for DNA barcoding [4–5]. Although ITS2 showed the highest resolving power among the most commonly used markers, it still lacks enough information to identify a broad range of species. It is estimated that no more than 70% of species can be successfully identified using this single locus [5]. Accordingly, there is an increasing tendency to combine multiple loci with the ITS/ITS2 region. However, increasing the number of nucleotides is expensive and time-consuming, and does not guarantee an improvement in the accuracy of tree building methods that are based heavily on substitute modeling [6]. To improve the situation, we need to obtain and use additional phylogenetic information about ITS2 without the addition of nucleotides.

Phylogenetic analyses of ITS2 have been based exclusively on its nucleotide sequences, but, until recently, few of these studies have taken into account its structure information. In cells, RNA activity is based on its secondary structure. Despite considerable variations in nucleotide sequences, the secondary structure of eukaryotic ITS2 has been shown to be highly conserved with four helices and some common motifs [7–9]. In phylogenetic analyses, homologous alignments are often based on nucleotide similarity. However, the fidelity of the alignments decreases above genus rank due to excessive sequence variability. The ITS2 secondary structure provides the key to this problem because the conserved nucleotide motifs help anchor multiple sequence alignments, and thus a more realistic picture of relationships at higher taxonomic levels can be produced [10–11]. In addition, the secondary structure is maintained through base-pair interactions between canonical base-pairs (AU, GC), non-canonical stable (GU), unstable (AC) and uncommon pairs (GA, AA) [12–13]. These paired and unpaired ITS2 structural states contain additional phylogenetic information not found in the primary sequence, so including this information can significantly improve phylogenetic estimates [14–15].

In recent years, the phylogenetic use of the ITS2 secondary structure has received increasing attention, and many analytical methods and related tools have been proposed. When using secondary structure phylogenetic information, one of the most crucial tasks is to work with reliable structures. Currently, RNA secondary structure can be predicted through free energy minimization and this is performed by the Mfold [16] or RNAstructure software [17]. In addition, a homology-based structure modeling approach, which uses the highly conserved zones and common motifs, improves the reconstruction of unknown RNA secondary structures [18]. Once a series of sequence-structure pairs are identified, accurate structure-based multiple alignment becomes a major determinant of analysis quality. However, standard multiple sequence alignment tools, such as Clustal X [19] or T-Coffee [20], cannot be used because they do not include structural information. Fortunately, programs, such as LocARNA [21], MARNA [22] and 4SALE [23], have been developed to perform automatic alignments based on RNA sequences and their structures. Furthermore, several methods have been developed that transform structure information into phylogenetic signals, such as the molecular morphometrics method [24–25], ProfDistS [26] and the 28 letter symbol coding method [27]. Although there are theoretical arguments for and against these methods or tools, there have been few accurate comparative analyses performed to evaluate to what extent secondary structure information benefits phylogenetic studies.

DNA barcoding, i.e. identification of species using standardized DNA regions across all possible forms of life, is a promising method for medical plant authentication [4], but has some critical drawbacks in practice. One of the major problems is taxon sampling. The multiple origin herb, derived from more than one species, is very common in traditional Chinese medical materials. Their substitutes or adulterants are often derived from closely related species but can come from unrelated species. For example, the traditional herbal remedy “Mu tong” is derived from at least four families: Aristolochiaceae, Lardizabalaceae, Ranunculaceae and Actinidiaceae, all of which are used as “Mu tong” in different areas of China. The first three families have even been included in the China Pharmacopeia [28]. Ever since the *Aristolochia manshuriensis* Kom. (Aristolochiaceae) “Mu tong” was found to contain highly toxic aristolochic acid and aristololactam [29–30], *Akebia* spp. (Lardizabalaceae) “Mu tong”, which do not contain toxic compounds, have been recognized as the legitimate “Mu tong”. However, Lardizabalaceae “Mu tong” include two very closely related species, which cannot be easily differentiated by ordinary herbalists, i.e. *Akebia quinata* (Houtt.) Decne. and *A*. *trifoliata* (Thunb.) Koidz. Therefore, the selection of barcode loci for authenticating traditional medicinal materials involves complex trade-offs between closely related species and distantly related species. The markers should be variable enough to be able to identify most close related species, but they should also be somewhat conservative in order to simplify PCR amplification and sequence alignment among distantly related species. This double standard presents a considerable challenge when choosing the best barcode [31].

Nucleotide variation in ITS is high, which is useful for discriminating between species that are closely related, but its conserved secondary structure can be used to align divergent sequences above the genus level. Therefore, ITS2 has been considered as a double-edged tool for eukaryotic evolutionary comparisons [32]. In this study, the ITS2 secondary structures of 30 accessions, representing 16 species and four families, were examined using the phylogenetic results from structure only data and combined sequence-structure data, respectively, to explore (1) whether sequence alignment is significantly improved if secondary structure information is included; and (2) how large is the improvement in species resolution by DNA barcoding if secondary structure information is included? Finally, we developed and tested the hypothesis that ITS2 secondary structure should be incorporated into DNA barcoding analysis. Thus, our results may provide new insights into ways of improving the current application of DNA barcoding by increasing species discrimination without the addition of nucleotides.

## Materials and Methods

### Taxon sampling and sequence acquisition

We sampled materials represent a broad taxonomic range with the aim to test the phylogenetic utility of secondary structure in higher taxonomic rank. In total, 30 accessions were used for this study, including six species of family Lardizabalaceae, three species of Aristolochiaceae, five species of Ranunculaceae and two species of Actinidiaceae, among which 14 accessions were retrieved from Genbank and 16 accessions were obtained from this study (Table 1). We extracted the total DNA from silica gel-dried leaves using the plant DNA Extraction Kit (Tiangen Biotech, Beijing, China). The PCR primer ITS5-ITS4 and their reaction conditions were followed from Baldwin [33] (S1 Fig). After purified with a TIANgel Midi Purification Kit (Tiangen Biotech, Beijing Co., LTD), the PCR amplification products were sequenced on a 96-capillary 3730XL DNA analyzer (Applied Biosystems, Foster City, CA, USA) using the PCR primers.

**Table 1:** Samples and their ITS2 Genbank accession numbers included in this study.

Species	Herb name	Family	Source	Genbank No.
*Akebia quinata* (Houtt.) Decne. 1	Mu tong	Lardizabalaceae	Jinggangshan, Jiangxi, China	KR025497
*Akebia quinata* (Houtt.) Decne. 2	Mu tong	Lardizabalaceae	Weihai, Shandong, China	KR025499
*Akebia trifoliata* (Thunb.) Koidz. 1	Mu tong	Lardizabalaceae	Laojun shan, Sichuan, China	KR025493
*Akebia trifoliate* (Thunb.) Koidz. 2	Mu tong	Lardizabalaceae	Genbank	GQ434615
*Akebia trifoliate* (Thunb.) Koidz. 3	Mu tong	Lardizabalaceae	Genbank	GQ434616
*Akebia trifoliate* (Thunb.) Koidz. 4	Mu tong	Lardizabalaceae	Huangshan, Anhui, China	KR025494
*Akebia trifoliate* (Thunb.) Koidz. 5	Mu tong	Lardizabalaceae	Jinggangshan, Jiangxi, China	KR025495
*Akebia trifoliate* (Thunb.) Koidz. 6	Mu tong	Lardizabalaceae	Genbank	KF022325
*Akebia trifoliate* (Thunb.) Koidz. 7	Mu tong	Lardizabalaceae	Tongcheng, Anhui, China	KR025496
*Akebia trifoliate* (Thunb.) Koidz. 8	Mu tong	Lardizabalaceae	Huating, Gansu, China	KR025498
*Akebia longeracemosa* Matsum.	——	Lardizabalaceae	Binzhou, Hunan, China	KR025500
*Holboellia parviflora* (Hemsl.) Gagnep.	——	Lardizabalaceae	Genbank	AY029795
*Stauntonia chinensis* DC. 1	——	Lardizabalaceae	Huangshan, Anhui, China	KR025501
*Stauntonia chinensis* DC. 2	——	Lardizabalaceae	Genbank	AY029787
*Sargentodoxa cuneata* (Oliv.) Rehder & E.H.Wilson	——	Lardizabalaceae	Huangshan, Anhui, China	KR025502
*Aristolochia kaempferi* Willd. 1	Huai Mu tong	Aristolochiaceae	Genbank	AM501928
*Aristolochia kaempferi* Willd. 2	Huai Mu tong	Aristolochiaceae	Genbank	AM501930
*Aristolochia manshuriensis* Kom. 1	Guang Mu tong	Aristolochiaceae	Ji’an, Jilin, China	KR025503
*Aristolochia manshuriensis* Kom. 2	Guang Mu tong	Aristolochiaceae	Jingyu, Jilin, China	KR025504
*Aristolochia clematitis* L.	——	Aristolochiaceae	Genbank	EF427951
*Clematis armandii* Franch.	Chuan Mu tong	Ranunculaceae	Kaixian, Chongqing, China	KR025505
*Clematis montana* Buch.-Ham. ex DC.	Chuan Mu tong	Ranunculaceae	Genbank	GQ434613
*Clematis buchananiana* DC. 1	Chuan Mu tong	Ranunculaceae	Midu, Yunnan, China	KR025506
*Clematis buchananiana* DC. 2	Chuan Mu tong	Ranunculaceae	Zhangmu,Xizang	KR025507
*Clematis argentilucida* (Levl. et Vant.) W. T. Wang	Chuan Mu tong	Ranunculaceae	Dali, Yunnan, China	KR025508
*Clematis finetiana* Levl. et Vaniot 1	Chuan Mu tong	Ranunculaceae	Genbank	GU732593
*Clematis finetiana* Levl. et Vaniot 2	Chuan Mu tong	Ranunculaceae	Genbank	JF714642
*Actinidia melanandra* Franch.	——	Actinidiaceae	Genbank	AF443211
*Actinidia arguta* (Siebold & Zucc.) Planch. ex Miq. 1	——	Actinidiaceae	Genbank	JF980325
*Actinidia arguta* (Siebold & Zucc.) Planch. ex Miq. 2	——	Actinidiaceae	Genbank	AY216736

### Secondary-structure prediction and sequence alignment

The ITS2 region was identified and delimited based on the Genbank annotation or Hidden Markov models (HMMs) which performed through the web server (http://its2.bioapps.biozentrum.uni-wuerzburg.de.) [18]. After Cut off the 3’ and 5’ termini of the ribosomal 5.8S and 26S rRNA, the complete ITS2 sequences were aligned with Clustal X [19] and adjusted manually using BioEdit 7.0.5 [34]. The primary sequence was folded through the MFOLD [16] at the default conditions and the structure with the minimum free energy was selected. The sequence-structure was aligned by MARNA web server (http://rna.informatik.uni-freiburg.de/MARNA/Input.jsp) [22] at the default setting with removing 2.0, arc breaking 1.0, arc mismatch 2.0, base deletion 1.5 and base mismatch 1.8. In addition, the sequences and structures were also automatically and synchronously aligned with 4SALE 1.5 [23] for comparison.

### Information coding and phylogenetic analysis

Sequence variation and Kimura 2-parameter (K2P) distance matrix were computed with MEGA 6.0 [35]. Unambiguous indels of the nucleotide sequence were treated as phylogenetic characters according to the simple indel coding method [36]. The alignment was performed by GapCoder [37], and the gap matrix was used in subsequent MP analyses.

We followed the 28-symbol method of Subbotin [27,38] and considered each of the four bases in helixes as six states (e.g., AA, AC, AG, AU, A-,-A), together with each of the four base states in loops as additional symbols [27]. After each state was designated as separate character a new structure information matrix was produced through RNAstat software [27]. The matrix of nucleotide sequence and secondary structure information was analyzed individually and combinedly using maximum parsimony (MP) methods. This analysis was performed through PAUP4.0b10 with the parameter setting as heuristic search of 1000 random addition replicates, tree bisection-reconnection branch swapping and the multrees option. Bootstrap analyses, based on 1000 replicates with 10 random additions per replicate were used to estimate the confidence of the clades. The nucleotide sequence data matrix and secondary structure matrix were analyzed separately and combinedly. The incongruence length difference test (ILD) [39] was used to assess information congruence between nucleotide sequence and secondary structure.

## Results

### ITS2 Secondary structure and the alignment of sequences

**Fig 1:**
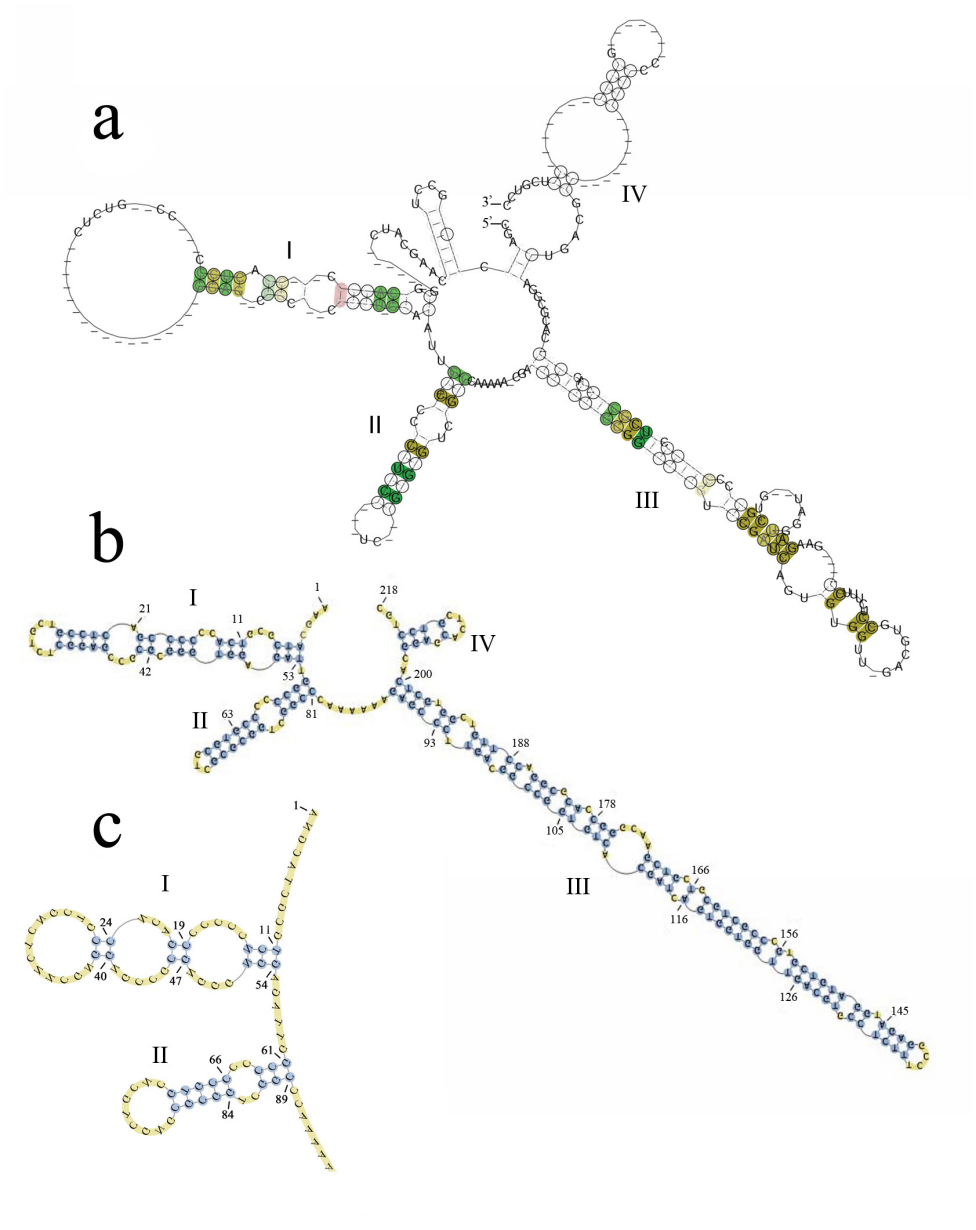
The predicted ITS2 secondary structure of the “Mu tong” taxa. (a) Consensus secondary structure model of the ITS2 region based on 30 sequences covering four families (Actinidiaceae, Aristolochiaceae, Lardizabalaceae and Ranunculaceae). The four helices, each with a stem–loop, are labeled I–IV. Compatible base pairs are colored and the degree of conservation over the whole alignment is indicated with different degrees of color saturation. (b) One of the example secondary structures of *Akebia quinata*. (c) The position of the strictly conserved base pair sites (highlight in blue circle) found in the 70% consensus ITS2 secondary structure model.

**Fig 2:**
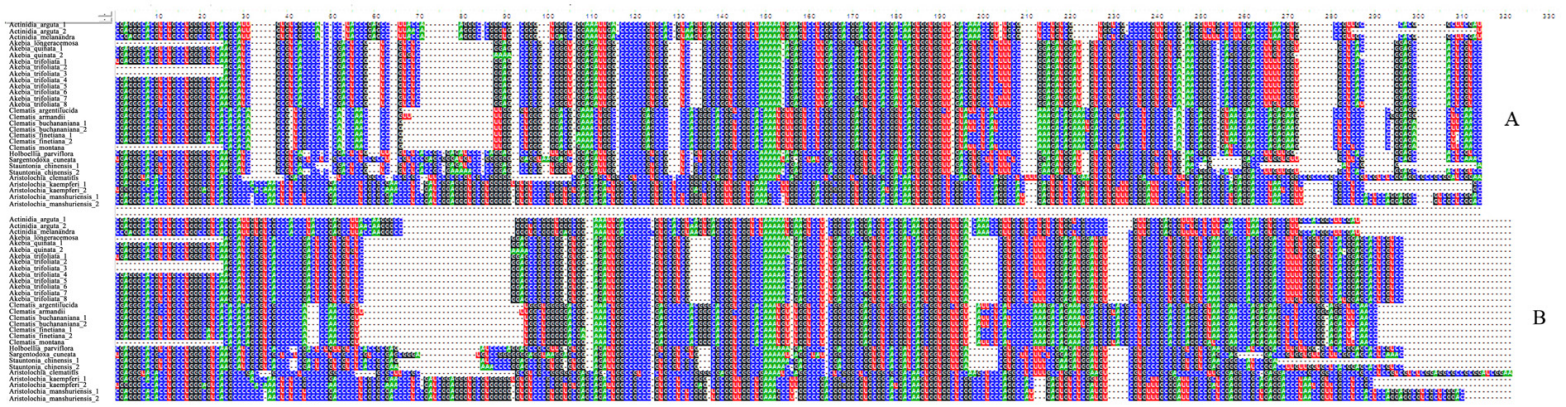
Comparison of multiple sequence alignments from different methods. a. alignment from MARNA with secondary structure guiding; b. alignment from Clustal X without secondary structure guiding.

ITS2 was aligned into 30 accessions from four families of “Mu tong” herbs. The accessions had a typical four-helix folding consensus structure, among which helix III was the longest, and helix IV was the most variable (Fig 1). Helix II was more stable, and contained a pyrimidine-pyrimidine bulge and a non-canonical U(T)-G base pair. In addition, some conserved motifs were also found in the helices, such as CUCC in helix I, C-G pairs in helix II, and GCGG and CGAUC in helix III (Fig 1a and 1b). The RNAstat analysis showed that the structure sequence ranged from 214 bp to 297 bp in length and the aligned length was 314 bp, among which 15 base pairs were strictly conserved (Fig 1c). The ITS2 nucleotide sequences were aligned, based on these shared structure constraints, and it is interesting that the alignment result was different from the Clustal X alignment, where only the primary homologous sequences were considered. There were differences between the number and position of the indels, and in the whole alignment length (Fig 2).

### Phylogenetic analysis

#### Comparison between sequence alignments, with and without structure guidance

**Fig 3:**
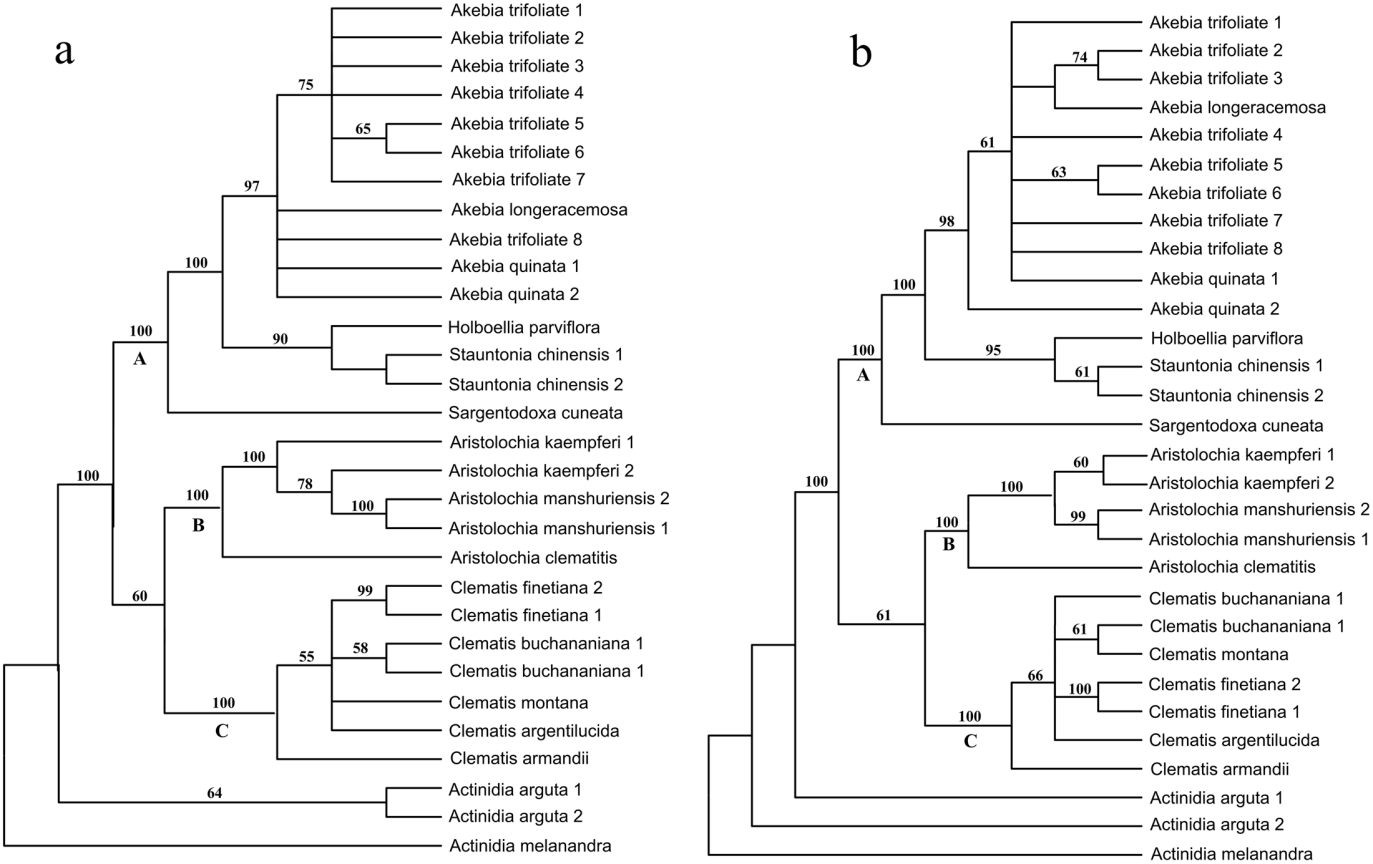
Comparison of MP trees inferred from sequences with different align methods. a. MP tree inferred from sequence alignment by Clustal X; b. MP tree inferred from sequence alignment by secondary structure information. Numbers on the branches indicate the bootstrap values of MP above 50%, numbers following a species name represent individual numbers.

The length of the alignment produced by Clustal X was 321 bp, and 37 indel-coding characters were added producing a total of 358 characters, among which, 262 were variable and 207 were parsimony informative. The MP analysis of the data resulted in 128 short trees, each with a length of 474 steps, a CI (consistency index) of 0.8143 and a RI (retention index) of 0.9364 (Table 2). We also performed an additional multiple alignment using MARNA, in which both the primary sequence and the secondary structure were taken into account. The results showed that there were 454 bp characters, which consisted of a 314 sequence alignment plus 140 additional indel-coding characters, among which 325 were variable and 222 were parsimony informative. In addition, the MP analysis of the data produced eight short trees, each with a length of 536 steps, a CI of 0.8190 and a RI of 0.9222 (Table 2). Clearly, sequence alignment using the secondary structure information did not change the sequence homoplasy, but greatly increased the number of parsimony informative characters (7.25%). The two alignments yielded topologically similar trees, both of which could be divided into four clades with the same phylogenetic relationships, e.g. the Actinidiaceae clade was in the basal position, and Aristolochiaceae and Ranunculaceae were close to each other and sisters to Lardizabalaceae (Fig 3). However, there were also some significant differences within the clades with regards to the placement of some specific species. For example, the two divergent *Aristolochia kaempferi* Willd. individuals were clustered together in a tree, based on structure-guiding alignment (Fig 3b). However, in the tree without structure-guiding alignment, *A*. *kaempferi* was not monophyletic because one of its individuals was nested within the *Aristolochia manshuriensis* Kom. clade (Fig 3a). Thus *A*. *kaempferi* cannot be successfully identified in the sequence alignments without structure guidance. In addition, support values in the tree based on structure-guiding alignments were higher than in the tree constructed without structure guidance. For example, there were 17 qualified support values (bootstrap ≥ 50%) in the three main clades of the tree based on structure-guiding alignments, while there were only 15 in the tree constructed without structure-guiding alignments (Fig 3, clades A–C).

**Table 2:** Statistics of phylogenetic analysis from different alignments.

Statistic	Sequence alignment 1 (guiding without structure)	Sequence alignment 2 (guiding with structure)	Secondary structure	Sequence 2+structure
Aligned length	321	314	315	629
Indel character	37	140	0	140
Total length	358	454	315	769
No. of variable characters	262	325	234	559
No. of parsimony information character	207	222	134	355
No. of shortest trees	128	8	90	61
Tree length	474	536	403	941
CI	0.8143	0.8190	0.8362	0.8236
RI	0.9364	0.9222	0.9241	0.9241

#### ITS2 secondary structure phylogenetic information

**Fig 4:**
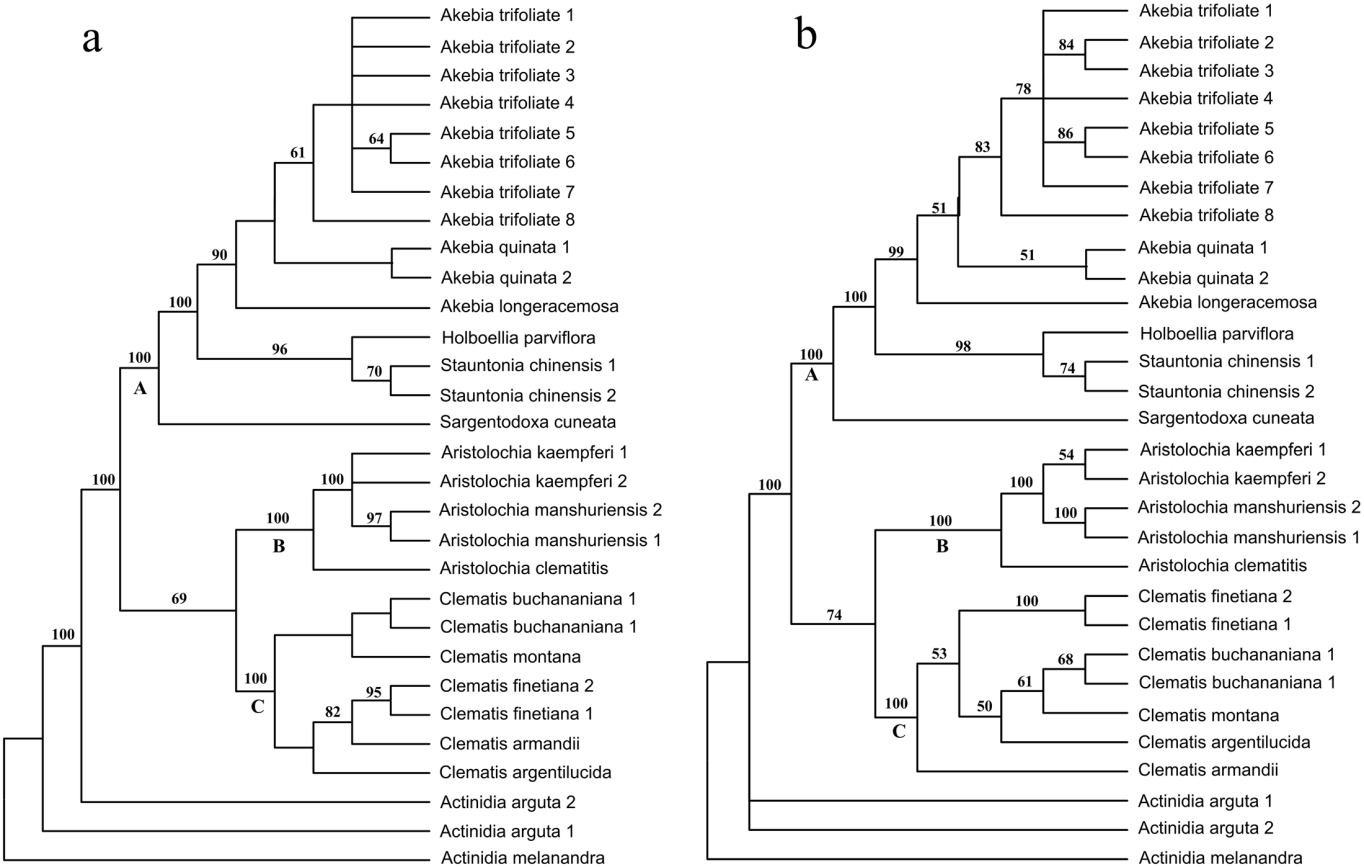
Comparison of MP trees inferred from different data sets. a. MP tree inferred from secondary structure coding information; b. MP tree inferred from the combined nucleotide sequences and secondary structure information. Numbers on the branches indicate the bootstrap values of MP above 50%, numbers following a species name represent individual numbers.

The ITS2 secondary structures were coded by RNAstat, which produced a total of 315 characters, among which 234 were variable and 134 were parsimony informative. A total of 90 short trees were produced by the MP analysis, each with a length of 403 steps, a CI of 0.8362 and a RI of 0.9241. The MP analysis yielded a major consensus tree that was topologically consistent with the tree constructed using just sequence alignment (Fig 4a versus Fig 3b), except for the phylogenetic position of *Clematis armandii* Franch. In the nucleotide sequence tree, *C*. *armandii* was placed in the basal position of clade C, while it nested deeply within this clade and was clustered together with *Clematis finetiana* Levl. et Vaniot in the tree constructed from secondary structure information (Fig 4a). The results of the ILD test showed no significant phylogenetic incongruence (*P* = 0.9384) between the sequence data and the structure data, so we combined both of them into a single dataset. As a result, the combined matrix consisted of 769 characters: 559 variable and 355 parsimony-informative characters. A total of 61 short trees were produced, which had a length of 941 steps, a CI of 0.8236 and a RI of 0.9214 (Table 2). Phylogenetic analysis of the combined data yielded a well-resolved tree. This tree was topologically congruent with the tree constructed by the sequence data, but with higher support values (Fig 4b).

#### Species discrimination

In the MEGA6 analysis, we assigned the samples to four clades according to family rank. The K2P distances showed that the mean distance within groups ranged from 1.80% (Ranunculaceae) to 17.74% (Aristolochiaceae). In contrast, the mean distances between groups ranged from 56.24% (Aristolochiaceae versus Lardizabalaceae) to 70.83% (Aristolochiaceae versus Actinidiaceae), and the largest distance was 73.43%. Although these high sequence divergences were beyond the level of reliable alignment by Clustal X, they were successfully aligned by structure guiding and yielded a robust MP tree. In this tree, the 30 individuals were divided into four clades. Three were monophyletic, well-supported, and corresponded to the Lardizabalaceae, Aristolochiaceae and Ranunculaceae families. This result showed that the herbs being described as “Mu tong” can be successfully identified through ITS2 using the family rank as the first-tier. In the Lardizabalaceae family (clade A), *A*. *quinata* (Mu Tong) and *A*. *trifoliata* (San Ye Mu Tong) were sister to each other. Individuals of the two species were each clustered together into a monophyletic group, which indicated that the herb “Mu tong” can also be successfully distinguished from its closest relatives. In addition, *Clematis buchananiana* DC. and *C*. *finetiana* Levl. et Vaniot (“Chuan Mu Tong”), *A*. *manshuriensis* (“Guan Mu Tong”), *A*. *moupinensis* and *A*. *kaempferi* (“Huai Mu Tong”), the most commonly used substitutes or adulterants, were all clustered together into monophyletic groups, which showed that they had been successfully identified.

## Discussion

It has been repeatedly shown that alignment quality may have a major impact on the final phylogenetic tree [40–42]. Some researchers have suggested that phylogenetic accuracy may depend more on alignment method than on the analysis method used for tree reconstruction [43–45]. This is especially true for highly distinct taxa, where the sequences are too divergent to be adjusted [46]. ITS2 is a promising DNA barcode because its fast substitution rate can provide sufficient resolving power for closely related species. This high variability, however, is also a major technical challenge when using ITS2 to barcode more divergent taxa, such as species-rich genera or taxa above genus level. In our study, discrimination between the traditional herbs that are often described as “Mu tong” required a large number of samples across four families. The MEGA6 results showed that the overall mean distance among samples was 43.43%, and the largest distance was 73.43%. We did not get an unambiguous alignment result because the sequence divergence was beyond the level that can be used to produce a reliable result [47]. Although the ITS2 nucleotide sequences substitute rapidly, their secondary structures are conserved by certain motifs. When the structure information has been taken into account during alignment, the new alignment is different from the original one, both in length and indel positions (Fig 2). As a result, two different MP trees were produced. The tree produced using structure-guiding alignment is considered the most accurate because it has a reliable topology and high support values. Therefore, we propose that the ITS2 secondary structure should be taken into account when undertaking DNA barcoding, particularly for extremely divergent taxa.

Theoretically, adequate discriminatory power needs highly variable barcode loci to provide more phylogenetic information, whereas the universality feature means that the loci need to be conservative enough for primer design. In practice, additional competitive characteristics, such as a long locus length to provide sufficient information versus short length for easy PCR amplification, also exist. These double standards have prevented the wide application of DNA barcoding. As a result, a tiered method has been proposed in plant DNA barcoding; that is, a conserved DNA region shared across all land plants provides resolution at a higher rank (e.g., genus or family) as a first-tier, and a more variable region provides resolution at the species level as a second-tier [48]. This method theoretically solves the dilemma. However, adding more loci is costly and time consuming in practice. The secondary structure consists of a number of paired regions. When a mutation occurs in one side of a pair, it is always correlated with substitution on the other side in order to retain the paired bond. This new substitution model, called compensatory base changes (CBCs) [49], is different from the currently widely used nucleotide substitution models, and can include additional information not found in the primary sequence [24,50]. In this study, we explored alternative methods of adding ITS2 secondary structures to increase phylogenetic information gathering without having to add nucleotides. The structure information in our study contained a considerable amount of phylogenetic information (Table 2), and produced nearly the same tree topology as the nucleotide data (Figs 3b and 4a). Although not all the nodes within our ITS2 analyses had robust support, our results do show equivalent levels of topological congruence when compared to the structure and sequence data. In addition, the ILD test results also showed that the two data sets were phylogenetically congruent, so we were able to combine them into a single data matrix. The combined data produced a well-resolved topology that was highly congruent with the nucleotide data tree, in which the bootstrap values were considerably improved by the addition of the structural information. As a result, the different “Mu tong” herbs were successfully identified using a single 250-bp ITS2 fragment.

Future work is needed to promote the phylogenetic use of ITS2 secondary structures. When applying secondary structures, one of the most essential tasks is to work with reliable data. Crystallography, in vitro structure probing (RNase footprint assay) and DMS accessibility test in vivo are the most reliable methods for reconstructing secondary structures [51–52]. However, these methods are not often applied in systematic studies. Alternatively, RNA secondary structure can be predicted through thermodynamic algorithms or homology modeling methods found in commercial software or online services, such as RNAstructure, Mfold and the ITS2-database. Unfortunately, the secondary structures from different folding models are not always strictly congruent. In addition, the lowest structure energy alone does not guarantee the best-model prediction [49]. Therefore, a better structure folding method and related software needs to be created. Another problem when using secondary structure to build phylogenetic trees is that the substitute mode in RNA secondary structure is more complex than in the nucleotide sequence. However, to date, no sophisticated evolutionary model for ITS has been created for phylogeny construction. Thus, this study only adapted a character-based MP analysis method so that we could avoid errors associated with the use of model-based algorithmic methods, such as NJ, Bayesian and PNJ [26]. However, sophisticated, phylogenetic, secondary structure level methods need to be created in the future.

## Conclusion

Traditional Chinese medicine is commonly composed of a complex mix of different species, including not only closely related species, but also more divergent species. DNA barcoding for medical plant authentication is promising method, but is limited by the imposition of single standard marker selection, because too variable or too conserved markers are both inappropriate in practice. Our study shows that the use of ITS2 sequences and structural data is a plausible method. The secondary structure information successfully sequence aligned the herb “Mu tong”, which contained four divergent families. In addition, our study identified the phylogenetic congruence of the ITS2 nucleotide sequences, and their secondary structure information. Although not all clades within our ITS2 results had robust support, the use of secondary structure information was successful because the additional characters increased bootstrap support and species resolution of “Mu tong” herbs. Thus, we recommend that ITS2 secondary structure information should be incorporated into future DNA barcoding analyses.

## Supporting Information

S1 Fig Structure of the ITS region of the nuclear ribosomal RNA genes and schematic location of primer used in this study. “ITS4” = TCCTCCGCTTATTGATATGC; “ITS5” = GGAAGTAAAAGTCGTAACAAGG. (Modified from Baldwin BG, 1995).
(TIF)
