# Supplementary figures and images for: ITS2 Secondary Structure Improves Discrimination between Medicinal “Mu Tong” Species when Using DNA Barcoding
Source: PLoS One. 2015 Jul 1;10(7):e0131185. doi: 10.1371/journal.pone.0131185 (PMC4488503; doi:10.1371/journal.pone.0131185)

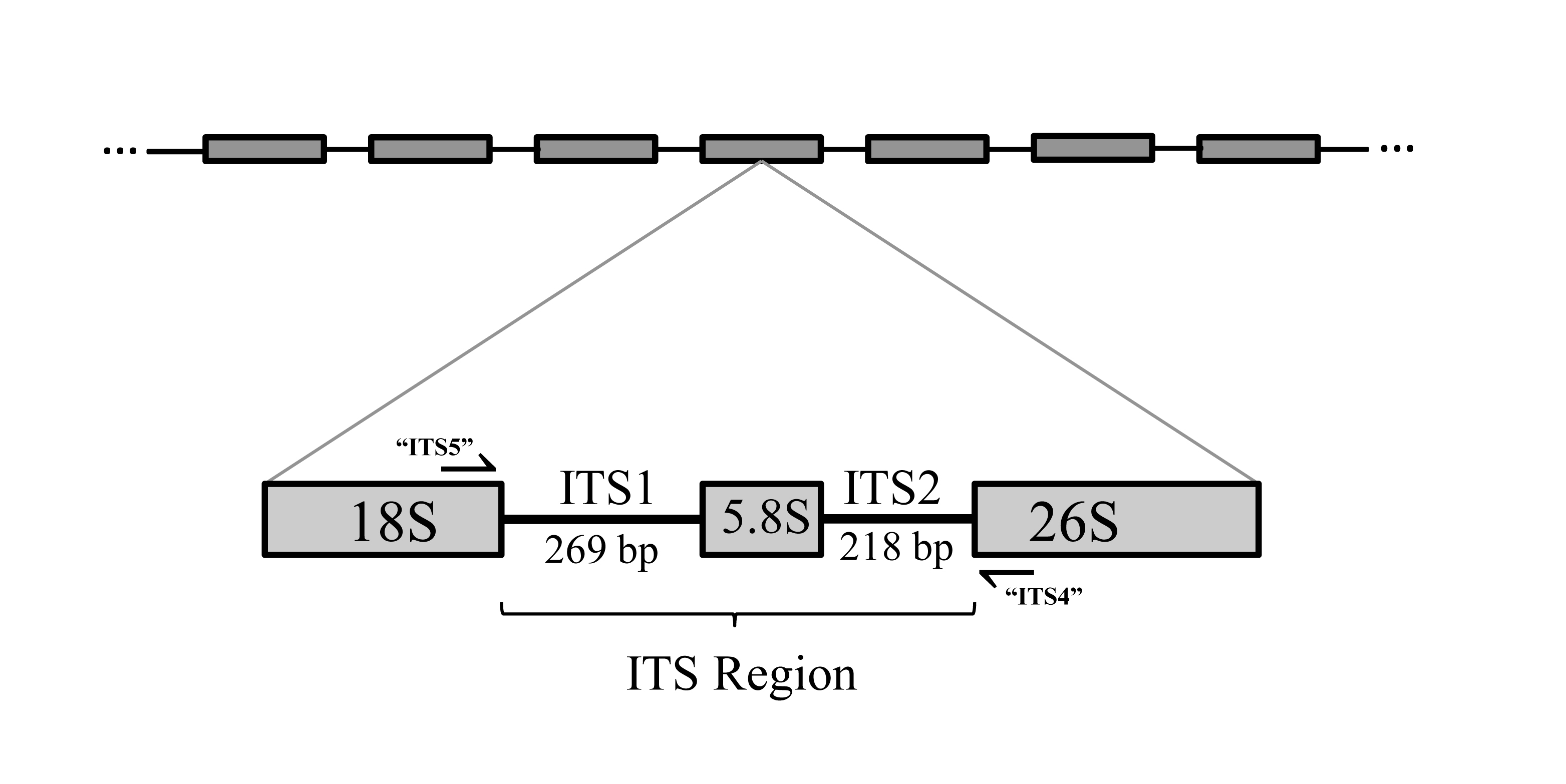

Supplement: S1 Fig — “ITS4” = TCCTCCGCTTATTGATATGC; “ITS5” = GGAAGTAAAAGTCGTAACAAGG. (Modified from Baldwin BG, 1995). (TIF) [file pone.0131185.s001.tif]
